# R-IDEAL: A Framework for Systematic Clinical Evaluation of Technical Innovations in Radiation Oncology

**DOI:** 10.3389/fonc.2017.00059

**Published:** 2017-04-03

**Authors:** Helena M. Verkooijen, Linda G. W. Kerkmeijer, Clifton D. Fuller, Robbert Huddart, Corinne Faivre-Finn, Marcel Verheij, Stella Mook, Arjun Sahgal, Emma Hall, Chris Schultz

**Affiliations:** ^1^Imaging Division, University Medical Center Utrecht, Utrecht, Netherlands; ^2^Department of Radiation Oncology, University of Texas MD Anderson Cancer Center, Houston, TX, USA; ^3^Department of Radiation Oncology, The Royal Marsden Hospital, The Institute of Cancer Research, London, UK; ^4^The University of Manchester, Institute of Cancer Sciences, Manchester Cancer Research Centre, The Christie NHS Foundation Trust, Manchester, UK; ^5^Department of Radiation Oncology, Netherlands Cancer Institute, Antoni van Leeuwenhoek Hospital, Amsterdam, Netherlands; ^6^Radiation Oncology, Sunnybrook Hospital, Toronto, ON, Canada; ^7^Clinical Trials and Statistics Unit, The Institute of Cancer Research, London, UK; ^8^Radiation Oncology, Froedtert and Medical College of Wisconsin, Milwaukee, WI, USA

**Keywords:** innovation, methodology, evaluation, MRI-guided linear accelerator, design

## Abstract

The pace of innovation in radiation oncology is high and the window of opportunity for evaluation narrow. Financial incentives, industry pressure, and patients’ demand for high-tech treatments have led to widespread implementation of innovations before, or even without, robust evidence of improved outcomes has been generated. The standard phase I–IV framework for drug evaluation is not the most efficient and desirable framework for assessment of technological innovations. In order to provide a standard assessment methodology for clinical evaluation of innovations in radiotherapy, we adapted the surgical IDEAL framework to fit the radiation oncology setting. Like surgery, clinical evaluation of innovations in radiation oncology is complicated by continuous technical development, team and operator dependence, and differences in quality control. Contrary to surgery, radiotherapy innovations may be used in various ways, e.g., at different tumor sites and with different aims, such as radiation volume reduction and dose escalation. Also, the effect of radiation treatment can be modeled, allowing better prediction of potential benefits and improved patient selection. Key distinctive features of R-IDEAL include the important role of predicate and modeling studies (Stage 0), randomization at an early stage in the development of the technology, and long-term follow-up for late toxicity. We implemented R-IDEAL for clinical evaluation of a recent innovation in radiation oncology, the MRI-guided linear accelerator (MR-Linac). MR-Linac combines a radiotherapy linear accelerator with a 1.5-T MRI, aiming for improved targeting, dose escalation, and margin reduction, and is expected to increase the use of hypofractionation, improve tumor control, leading to higher cure rates and less toxicity. An international consortium, with participants from seven large cancer institutes from Europe and North America, has adopted the R-IDEAL framework to work toward coordinated, evidence-based introduction of the MR-Linac. R-IDEAL holds the promise for timely, evidence-based introduction of radiotherapy innovations with proven superior effectiveness, while preventing unnecessary exposure of patients to potentially harmful interventions.

## Background

The pace of innovation in interventional oncology is high, and the window of opportunity for evaluation is narrow. Financial incentives, industry pressure, and patients’ demand for “high-tech” treatments have led to widespread implementation of innovations without robust clinical evidence of improved outcomes (1). This is also the case in radiation oncology, where intensity-modulated radiotherapy (IMRT) was widely implemented before robust evidence of clinical superiority over standard treatment was generated, and for proton therapy, where high level (randomized) evidence of clinical superiority is lacking (2–4).

Ideally, before implementation, all radiotherapy innovations should be evaluated in a systematic manner, where—ultimately—one compares effectiveness of the new technology against the standard treatment and evaluates whether it can lower toxicity, improve local control and survival, or save time or costs.

Until now, the phase 1, 2, and 3 trial framework has been applied to evaluate safety and efficacy of new radiotherapy interventions. However, this may not be the most efficient and desirable framework for assessment of radiotherapy innovations (5–7). For pharmaceuticals in oncology, phase 1 (i.e., dose escalation) studies are typically performed among metastasized patients with limited life expectancy. This is not an optimal choice in the radiotherapy setting, where radiation-induced toxicities occur to up to a year or more and will be missed if only patients with limited life expectancy are included. Also, radiation dose, and dose to the organs at risk (OARs) in particular, cannot be standardized due to variation in anatomical disease distribution (5). When investigating a new radiotherapy treatment for early efficacy and toxicity (phase 2), a single-arm study is rather uninformative due to the high variability in patient and treatment-related factors that affect outcome, such as tumor size, exact tumor location, radiation tolerance of the OARs, and (cardiovascular) co-morbidity. It has, therefore, been proposed to test early efficacy and toxicity of radiotherapy interventions in a randomized fashion (5).

Radiotherapy interventions are complex interventions, the evaluation of which is complicated by dependence on operator and team, learning curves and differences in levels of experience, and quality control. From this perspective, they resemble surgical innovations (8). In 2009, the Balliol consortium proposed recommendations for the assessment of surgical innovations, based on a five-stage description of the surgical development process called IDEAL (Idea, Development, Exploration, Assessment, and Long-term evaluation) (9). IDEAL aims for undelayed clinical implementation of superior interventions, without exposing patients to unnecessary risks. Recently, an IDEAL approach was proposed for systematic evaluation of medical devices (IDEAL-D) (10). In the present paper, we describe an IDEAL-based stepwise approach for evaluation of radiotherapy innovations (R-IDEAL), using examples from a recent innovation in radiation oncology—the MRI-guided linear accelerator (MR-Linac).

## MRI-Guided Linear Accelarator

In 1999, researchers at the UMC Utrecht conceptualized the Magnetic Resonance-guided linear accelerator (MR-Linac) (11). The MR-Linac system comprises a 6-MV Linac (Elekta Instrument AB, Stockholm, Sweden) mounted on a ring around a 1.5-T MRI scanner (Achieva, Philips Healthcare, Amsterdam, the Netherlands) and an online adaptive radiotherapy planning system (11) (Supplementary material). The system is designed to image patients and irradiate them simultaneously. The accelerator and MRI are designed to be magnetically decoupled so that the MR images are not distorted by the presence of magnetized accelerator components, and the operation of the accelerator is not hampered by the magnetic field. A series of MR sequences can be performed to produce pre-, during- and post-treatment images. Once the MR-Linac is fully developed, the pre- and post-treatment MRI will include morphological and functional images. The system provides real-time soft-tissue imaging during treatment delivery and allows tumor tracking for high-precision targeting (12). Theoretically, more precise targeting enables dose escalation leading to improved local control and survival. Higher precision permits the use of smaller margins and may result in lower toxicity. MR-Linac also has the potential to allow non-invasive ablative radiotherapy treatment for tumor sites where stereotactic ablative radiotherapy is not routine practice.

For the purpose of systematic evaluation of innovations in radiation oncology, including MR-Linac, we adapted the IDEAL framework. Here, we took into account that, in contrast to surgery, radiotherapy innovations often require simultaneous implementation of other technologies (i.e., hardware, software). Also, unlike surgery, effects of radiotherapy can be accurately modeled (13). R-IDEAL has been adopted by an international consortium to work toward coordinated and evidence-based introduction of the MR-Linac (14). The MR-Linac consortium was set up in 2012 and consists of collaborators of seven large cancer institutions in Europe and North America and two companies (Elekta and Philips). Nine tumor-site working groups are currently identifying clinical scenarios where MR-Linac technology (theoretically) could be of the most benefit for patients in terms of higher cure rates and/or lower toxicity. Each group works toward these goals by designing studies according to the adapted IDEAL framework (R-IDEAL).

## The R-Ideal Stages

R-IDEAL closely resembles the original IDEAL recommendations (9). Since most radiotherapy innovations may be used at different disease sites and for different indications (i.e., margin reduction, dose escalation, etc.), some R-IDEAL steps may need to be repeated for the same tumor site. Some stages may also overlap between tumor sites, making some steps redundant for certain indications.

### Stage 0: Radiotherapy Predicate Studies

Stage 0 covers all preparatory work needed before the innovation is ready for clinical use (Table 1). We differentiate two types of Stage 0 studies. Some Stage 0 studies address the issue on *how* the innovative treatment will be delivered, including development of contouring atlases and new coils, assessment of inter-operator variation, and optimization of imaging sequences.

An example of a Stage 0 study for MR-Linac, is a study in which we compare inter-observer reproducibility of MR delineation versus CT delineation of the gross tumor volume in oesophageal cancer. Planning studies assess the effect of the 1.5T magnetic field on dose distribution.

**Table 1:** **R-IDEAL stages for systematic evaluation of innovations in radiation oncology**.

	Radiotherapy—predicate studies	Idea	Development	Exploration	Assessment	Long-term evaluation
	
	Stage 0	Stage 1	Stage 2a	Stage 2b	Stage 3	Stage 4
Purpose	Answer the following questions before actual use of the innovation for treatment of patients How to use the innovation (software, coils needed)? Why and in whom to use the innovation?	First time use of the innovation for treatment delivery in men	Technical optimization of the innovation for treatment delivery	Provide proof of early clinical effectiveness and safety of the innovation	Formal comparison of innovation against standard treatment	Long-term outcomes of the innovation, post-marketing, and surveillance

Outcomes	– MR sequences, dedicated coils, etc. – Inter-rater reproducibility – Treatment strategies, patient selection	Proof of concept	Technical improvements, feasibility, and safety	Early effectiveness – toxicity – tumor response – local recurrence (with spacious information)	Effectiveness compared to standard treatment – (disease-free) survival – recurrence – toxicity – PROMs, CTC-PRO, – Cost effectiveness	Long-term toxicity, long-term (disease-free) survival, rare side effects, Patient-Reported Outcomes

Study design	Phantom studies, delineation studies, planning studies, model-based studies	Structured case report	Prospective small uninterrupted case series	Prospective study with preferably randomized component: RCT; cmRCT; random allocation of limited available treatment slots to eligible patients; Comparison with matched (historical) controls	RCT, cmRCT, registry-based trial	Prospective registries, including all patients treated with the innovation

Example	Inter- and intraobserver reproducibility of MR versus CT delineation of the gross tumor volume in esophageal cancer	1a. MR-Linac delivered conventional palliative radiotherapy for metastatic esophageal cancer 1b. MR-Linac delivered neoadjuvant chemoradiation with reduced PTV margins for Stage IIB–IIIB esophageal cancer	Small prospective cohort study of 10–20 patients with Stage IB–IIIC esophageal cancer undergoing MR-Linac delivered neoadjuvant chemoradiation using standard fractionation (23–28 times 1.8 Gy) with reduced PTV margins, with the aim to optimize technology and provide additional evidence of safety	A randomized study in patients with Stage IB–IIIC esophageal cancer who will be treated with neoadjuvant chemoradiation using standard fractionation (23–28 times 1.8 Gy), with reduced PTV margins delivered on the MR-Linac versus treatment with conventional margins delivered on a linear accelerator. Outcomes include pCR, 2-year mortality, recurrence, and toxicity	Multicenter RCT comparing mid- and long-term survival, toxicity, quality of life, and cost-effectiveness of MR-Linac delivered standard fractionation with reduced PTV margins versus standard radiotherapy of patients with Stage IB–IIIC esophageal cancer	The MR-Linac consortium has committed to registering technical data, patient and tumor characteristics, imaging, treatment, local control, survival, and toxicity in a collaborative registry

*Adapted from McCulloch et al. (9)*.

*For some innovations in radiation oncology, two levels of Stage 1 can be identified. In the case of MR-Linac, in *Stage 1a*, the technique is used for the first time to deliver standard treatment (standard dose and fractionation) in 1 to 3 patients to test the feasibility of using a linear accelerator in a magnetic field at a certain tumor site. In Stage 1b, for the first time, a different treatment strategy (e.g., dose escalation or margin reduction) is applied at a specific tumor site*.

*Stage 2a may follow Stage 1a (technical optimization of the innovation for standard dose delivery and fractionation) and Stage 1b (technical optimization of the innovation for a new indication, for example, dose escalation or margin reduction)*.

*RCT, randomized controlled trial; cmRCT, cohort multiple randomized controlled trial*.

Other Stage 0 studies answer the question on *why* the technique is used. What is the expected benefit of using this new technique instead of the standard treatment (higher cure rates, lower toxicity, or both)? Which patient categories and tumor types are expected to benefit most? For this purpose, the effect of dose escalation on tumor response and/or the effect of margin reduction on healthy tissue toxicity is modeled (13). This allows prediction at which tumor sites and with which strategies the new innovation is likely to be of most benefit. Also, these modeling studies give an indication whether it is safe for the technique to be translated to clinic. Early health technology assessment studies may indicate which treatment strategies can be expected to be cost effective or how much more effective the innovation needs to be to justify the extra costs (15).

Another example of a Stage 0 study in silico planning studies will compare dose distributions to OAR between MR-Linac and conventional dose distribution, in order to predict the potential reduction in radiation-induced side effects by reduction of the PTV margin.

The number of patients included in Stage 0 depends on the type and aim of the study. Development of coils may only require healthy volunteers, MRI sequence optimization and reproducibility studies typically include around 20–40 patients, while some other studies, like model-based approach, require no volunteers or patients at all. Outcome of Stage 0 include reports on inter-observer reproducibility, delineation studies, modeling studies, and early health technology assessment studies.

### Stage 1: Idea

In this Stage, the innovation is used for the first time at a certain tumor site in a patient (Table 1). Stage 1 demonstrates feasibility and absence of unexpected detrimental effects. Patients in Stage 1 are highly selected and perfect candidates for the intervention. Successful completion of the procedure, as well as unexpected technical and clinical complications will be reported as a case report. Systematic reporting of all results of Stage 1 studies allows groups to learn from each other and prevent similar (technical) complications. Since unexpected toxicity may occur up to months after the intervention, depending on OARs, a follow-up time needs to be incorporated before proceeding to Stage 2a. After Stage 1, it may be necessary to go back to Stage 0 to address technical problems.

### Stage 2a: Development

After Stage 1 has shown technical feasibility, without major unexpected toxicities or complications, the technique enters Stage 2a (development) (Table 1). Stage 2 aims to refine the technique and to optimize the work-flow. Stage 2a includes 10–30 patients. Outcomes of this Stage are technical feasibility and safety.

In Stage 2a, planned and unplanned technical modifications can be made. Details and timing of these modifications are reported, providing other groups with potentially helpful information for the use of the innovation at other tumor sites. All patients are reported consecutively, including unsuccessful or canceled interventions and reasons for that. Reports contain inclusion criteria, proportion of eligible patients treated with the new innovation, clear descriptions of each procedure, technical modifications made (with timeline), and toxicity. Stage 1 and Stage 2a may be submitted for ethical approval at the same time, with predetermined stopping rules, safety monitoring, and rules for when to move on to the next Stage or reiteration of the previous Stage.

For some radiotherapy innovations, two levels of Stage 1 and Stage 2a can be identified. In the case of MR-Linac, in Stage 1a, MR-Linacs used for the first time to deliver standard dose and fractionation in 1 to 3 patients to test the feasibility of using a linear accelerator in a magnetic field at a certain tumor site.

An example of a Stage 1a study is a study where MR-Linac delivered palliative radiotherapy using conventional margins and fractions (5 times 4 Gray) is administered to 1-3 patients with metastatic esophageal cancer to demonstrate feasibility of standard treatment of MR-Linac

After MR-Linac has been used for the first time for standard dose delivery and fractionation (Stage 1a), technical refinement and optimization of the work-flow for standard dose delivery and fractionation will be performed in Stage 2a.

An example of Stage 2a is a small prospective cohort study including 10-20 patients with metastatic esophageal cancer who receive MR-Linac-delivered palliative radiotherapy using conventional margins and fractions (5 times 4 Gray)

After technical details and workflow of the technique for standard treatment delivery have been optimized, the assessment procedure proceeds to Stage 1b. In Stage 1b, for the first time, a different treatment strategy (dose escalation or margin reduction) is applied at a specific tumor site. Stage 1b, like Stage 1a, includes 1–3 highly selected patients, who are perfect candidates for the intervention.

An example of a Stage 1b study is a study including 1-3 patients with stage IB-IIIC esophageal cancer who undergo MR-Linac delivered neoadjuvant chemoradiation using standard fractionation (23-28 times 1.8 Gray) with reduced PTV margins

After a proof of concept for this innovative approach has been demonstrated, the technique re-enters Stage 2a, in which additional modifications are made to further optimize work-flow and technology for innovative treatment delivery.

An example of a Stage 2a study is a small prospective cohort study of 10-20 patients with stage IB-IIIC esophageal cancer who undergo MR-Linac delivered neoadjuvant chemoradiation using standard fractionation (23-28 times 1.8 Gray) with reduced PTV margins

### Stage 2b: Exploration

After the main technical details have been worked out, the focus of the R-IDEAL assessment procedure shifts from technical optimization to evaluation of clinical effectiveness. In Stage 2b, evidence of early efficacy is provided (Table 1). Outcomes in Stage 2b depend on tumor site and may include clinical and pathological tumor response, toxicity, early tumor recurrence, and (disease-free) survival. Spatial information is collected in case of early local recurrence. Recurrence in the center of the tumor suggests under-dosing while recurrence at the periphery of the tumor suggests too narrow margins. Studies in Stage 2b typically have a follow-up period of 18–24 months. Based on the outcomes of Stage 2b, investigators may need to go back to Stage 2a, to address technical issues, or even Stage 0, to improve treatment strategy and patient selection.

Stage 2b preferably adopts a randomized design, such as a randomized controlled trial. The reason for this is that many factors may affect efficacy and toxicity, such as exact tumor size, anatomic site, radiation tolerance of OARs, and comorbidities. Usually, the number of treatments that can be delivered using the new innovation is limited when the technique is first introduced (limited amount of treatment slots). This provides a unique opportunity for randomized evaluation, as the limited available treatment slots may be randomly allocated to eligible patients. If, for example, in a certain week two treatment slots for a certain tumor site are available, while there are five eligible new patients, two patients will be randomly selected for treatment on the MR-Linac, while the other three receive standard care. In the next week, with two available treatment slots and three eligible new patients, two patients are randomly allocated to MR-Linac treatment. This period-stratified random sampling of a predetermined fixed number of patients from a variable sized “dynamic” pool will bring a randomized component in the comparison, reduce the potential for selection by indication, and maximize the use of the available slots. If, due to time and financial constraints, randomization is not feasible, prospective registries or cohort studies, with matched (historical or contemporary) controls, may be good alternatives.

In Stage 2b, quality control measures will be reported to provide clear understanding of the accuracy with which the procedure has been performed.

An example of a Stage 2b study is a randomized trial, including patients with stage IB-IIIC esophageal cancer. The intervention arm will be treated on MR-Linac and receive neoadjuvant chemoradiation using standard fractionation (23-28 times 1.8 Gray) and reduced PTV margins. The control arm is treated on a standard linear accelerator and receives neoadjuvant chemoradiation using standard fractionation (23-28 times 1.8 Gray) with conventional margins. Both groups are compared in terms of pathological complete response (pCR) rates, 2 year mortality, early recurrence, and toxicity.

### Stage 3: Assessment

When Stage 2b has provided early evidence that the innovation may accomplish the radiation treatment more effectively or with fewer side effects, the treatment will be compared against standard therapy (Table 1). The randomized control trial (RCT) remains the design of choice. However, RCTs may be challenging due to recruitment difficulties, strong doctor and patient preferences, different perceptions of equipoise, and limited capacity of the new equipment (16, 17). Alternative designs may be considered, including the registry-based RCT (18) or the cohort multiple RCT (19). In these designs, prospective cohorts or registries serve as facilities for (multiple) pragmatic randomized comparisons of new interventions against standard treatment.

Outcomes of Stage 3 include (disease-free) survival, recurrence, tumor response, and toxicity and cost-effectiveness. In addition to these outcomes, the perspective of the patient (i.e., quality of life, fatigue, workability) provides valuable and complementary input, and a more robust appreciation of patients’ symptoms (20). The patient-reported outcomes version of the Common Terminology Criteria for Adverse Events (PRO-CTCAE) intertwines the patient perspective directly into the AE reporting system (21, 22) and gives an accurate estimation of treatment-induced toxicity.

Stage 2b and 3 may be submitted as a single protocol, with clear definition of stopping rules. When Stage 3 shows negative or neutral results for the indication under study, introduction into regular care is not justified (not evidence-based).

An example of a Stage 3 study is a multicenter RCT including patients with stage IB-IIIC esophageal cancer. The intervention arm will be treated on MR-Linac and receive neoadjuvant chemoradiation using standard fractionation (23-28 times 1.8 Gray) and reduced PTV margins. The control arm is treated on a standard linear accelerator and receives neoadjuvant chemoradiation using standard fractionation (23-28 times 1.8 Gray) with conventional margins. Both groups are compared in 5 year survival, toxicity, quality of life and cost effectiveness.

### Stage 4: Long-term Study

Prospective registries will be set up to capture technical, clinical, treatment, and outcome data of patients who underwent the new intervention, allowing large-scale evaluation of long-term effectiveness and rare side effects (Table 1). Also, variations in outcomes between institutions can be assessed, which may be indication of differences in quality assurance or patient selection. All patients who have been exposed to the innovations, starting at Stage 1, will enter the registry. The registry is setup in such a way that it also allows data exchange between institutions who have invested in the innovation, using high standards for data interoperability, quality control, privacy, and data ownership (23).

The MR Linac consortium has committed to registering technical data, patient and tumor characteristics, imaging, treatment, local control, survival, and toxicity in a collaborative registry.

## Discussion

We adapted the IDEAL framework to fit the radiotherapy setting and encourage systematic evaluation of innovations in radiation oncology. Innovations in radiation oncology vary from minor technical adaptations, i.e., new MRI sequences or new coils, to major innovations with large potential therapeutic and societal impact. History has shown that technical innovations in the areas of surgery and radiotherapy run a high risk of being implemented without rigorous evaluation. Introducing a new technology implicates changes in imaging modalities and/or workflow, need for new software, quality assurance, training of the multidisciplinary team, and development of team expertise. New technologies frequently imply significant investment for the developer and the user. Therefore, evaluation for radiotherapy innovations is more complex than pharmaceutical innovations, where investment on the whole is only made by the developer. The need for users to have returns on their investment, both financial and professional, frequently compromises the evaluation of the technology and can fuel non-evidence-based use. The use of protons has increased exponentially in the last few years. However, theoretical advantages of proton therapy have not yet been confirmed in randomized trials. A study among 27,647 Medicare recipients with prostate cancer (553 of whom were treated with proton therapy) showed that although proton therapy was substantially more costly, there was no measurable reduction in toxicity at 12 months posttreatment (24). Nonetheless, proton therapy is being offered to a growing number of patients, often on their demand. Similarly, IMRT is widely used, although its exact benefit is not known for all clinical indications. Several studies have shown reduced toxicity for head and neck (4, 25) and breast cancer (26). For many clinical indications, superiority of IMRT has not been proven, or was proven only after IMRT was widely adopted (27). Also, the impact of an increase in the volume proximal to the tumor receiving a low radiation dose on the induction of secondary tumors has been insufficiently investigated.

Like IMRT, proton therapy, and MR-Linac, most technical innovations in radiation oncology can be used for different indications and at different sites. The R-IDEAL framework accelerates identification of areas where the new technology is likely to be of most benefit, and areas where the new technology is not feasible (Stage 1, 2a), not (more) effective (than standard therapy) (Stage 2b, 3) or associated with long-term adverse effects (Stage 4). Early abandonment of development of new technologies for unsuitable indications, and prevention of implementation, will reduce costs and harms and allow the focus to be shifted to areas where the technique is more likely to provide benefit. This is in the interest of vendors, users, patients and society. Finally, the R-IDEAL framework is not (primarily) intended as a mechanism to support regulatory clearance, but rather as a process and strategy to iterate an innovative technology. While the regulatory process is independent from the R-IDEAL process, there are some common elements between the two. In the case of MR-Linac, a good example is the development of imaging sequences which is part of R-IDEAL and is also necessary for 510(k) clearance.^1^

In conclusion, the R-IDEAL framework was adapted to fit the radiation oncology setting in order to facilitate systematic evaluation of innovations in radiation oncology. R-IDEAL holds the promise for timely, evidence-based introduction of radiotherapy innovations with proven superior effectiveness, while preventing unnecessary exposure of patients to potentially harmful interventions.

## Author Contributions

HV conceived of the study, participated in its design and coordination, and drafted the manuscript. HV, LK, CF, RH, CF-F, SM, and EH substantially participated in its designing and coordination of the study and drafting the manuscript. HV, LK, CF, MV, AS, and CS participated in coordination of the study. All the authors read, gave feedback, and approved the final manuscript.

## Conflict of Interest Statement

Elekta and Philips are members of the MR-linac Consortium. The other authors declare no conflict of interest.

## Footnotes

^1^Available from: http://www.fda.gov/MedicalDevices/ProductsandMedicalProcedures/DeviceApprovalsandClearances/510kClearances/
