# Supplementary material for: R-IDEAL: A Framework for Systematic Clinical Evaluation of Technical Innovations in Radiation Oncology
Source: Front Oncol. 2017 Apr 3;7:59. doi: 10.3389/fonc.2017.00059 (PMC5378068; doi:10.3389/fonc.2017.00059)
Supplement: Supplementary file 1 [file Image_1.PDF]

Supplementary material

MRI guided linear accelerator: Linear accelerator (gantry) around a 1.5 Tesla MRI scanner .

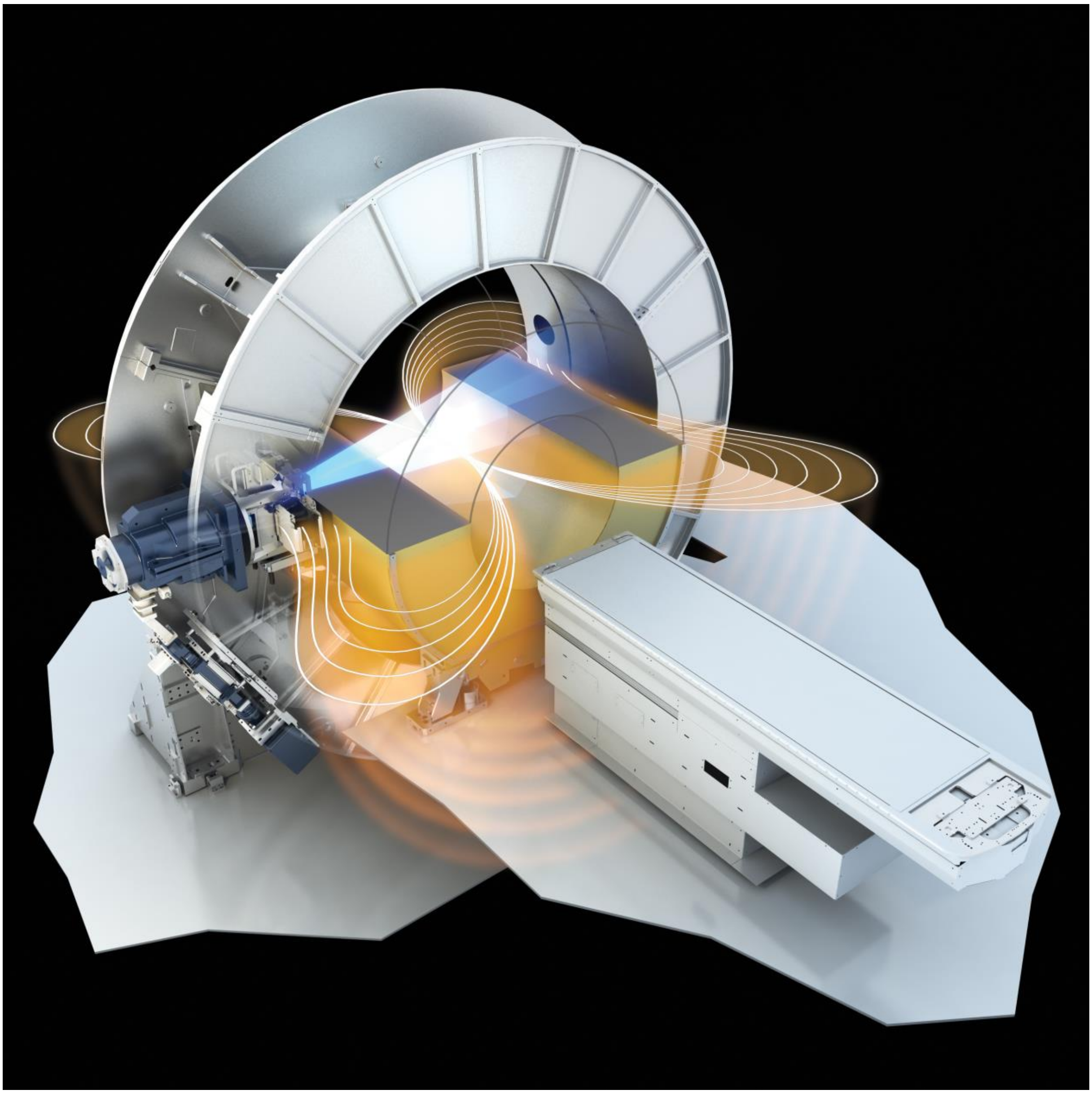

With courtesy of Elekta AB, Stockholm Sweden
